# Contribution of Extracranial Injuries to GOSE Scores after Traumatic Brain Injury TBI: A TRACK-Traumatic Brain Injury Study

**DOI:** 10.1089/neu.2024.0421

**Published:** 2025-09-12

**Authors:** Nancy Temkin, Jason Barber, Joan Machamer, Gabriela Sugar, Molly Rose Morrissey, Kim Boase, Evan Zahniser, Yelena G. Bodien, Joseph T. Giacino, Michael A. McCrea, Lindsay D. Nelson, Murray B. Stein, Sabrina Taylor, Claudia Robertson, David Okonkwo, Geoff Manley, Sureyya Dikmen, the TRACK-TBI Investigators

**Affiliations:** ^1^ Department of Neurological Surgery, University of Washington, Seattle, Washington, USA.; ^2^ Department of Biostatistics, University of Washington, Seattle, Washington, USA.; ^3^ Department of Neurological Surgery, University of California San Francisco, San Francisco, California, USA.; ^4^ Seattle Division, VA Puget Sound Healthcare System, Seattle, Washington, USA.; ^5^ Department of Physical Medicine and Rehabilitation, Spaulding Rehabilitation Hospital, Harvard Medical School, Charlestown, Massachusetts, USA.; ^6^ Department of Neurology, Massachusetts General Hospital, Harvard Medical School, Boston, Massachusetts, USA.; ^7^ Departments of Neurosurgery and Neurology, Medical College of Wisconsin, Milwaukee, Wisconsin, USA.; ^8^ Department of Psychiatry and Herbert Wertheim School of Public Health, University of California San Diego, San Diego, California, USA.; ^9^ Brain and Spinal Injury Center, San Francisco, California, USA.; ^10^ Department of Neurosurgery, Baylor College of Medicine, Houston, Texas, USA.; ^11^ Department of Neurological Surgery, University of Pittsburgh School of Medicine, Pittsburgh, Pennsylvania, USA.; ^12^ Department of Rehabilitation Medicine, University of Washington, Seattle, Washington, USA.

**Keywords:** extracranial injury, Glasgow Outcome Scale Extended, global outcome, multiple trauma, TRACK-TBI, traumatic brain injuries

## Abstract

The Glasgow Outcome Scale Extended (GOSE) is the most widely used outcome measure for hospital-based studies of traumatic brain injury (TBI). The GOSE may be administered several ways, the choice depending on the purpose of the research. In this investigation, we evaluated the effect of administering the GOSE to collect functional disability attributed to all injuries sustained (GOSE-All) or excluding the impact of extracranial injuries (GOSE-TBI). We examined the differences in reported disability between the two administration methods at 2 weeks, 3 months, 6 months, and 12 months after injury. Data are summarized from 2288 individuals who were enrolled in the prospective observational Transforming Research and Clinical Knowledge in TBI (TRACK-TBI) cohort study. The distribution of scores is summarized by time after injury, brain injury severity, and extracranial injury severity. Dichotomizing the GOSE varying ways, differences in the prevalence of unfavorable outcomes for GOSE-All versus GOSE-TBI range from none to 42 percentage points. Discrepancies in disability captured by GOSE-All and GOSE-TBI decrease with greater TBI severity, no serious extracranial injuries, and longer time post-injury. It is important for researchers, given the aims of their studies, to decide in advance whether GOSE classification should be based on the effects of all injuries sustained or excluding the effects of extracranial injuries so as to emphasize the effects of the brain injury, as well as how disability due to emotional consequences of injury and other circumstances will be scored. Instructions to the respondent and outcomes examiner need to be clear about what causes of disability are to be included. The TBI Common Data Elements should include information that reflects the method that was used to collect the GOSE data and data repositories should disclose which data collection method was used for a given study.

## Introduction

Approximately 2.5 million people in the United States sustain a traumatic brain injury (TBI) annually, making it one of the leading causes of death and disability nationally.^1^ TBI that occurs each year has an estimated lifetime cost of $758 billion, bringing this disease to the forefront of public health concerns.^2^

The Glasgow Outcome Scale (GOS) or the GOS-Extended (GOSE)^3^ is the most widely used outcome measure for hospital-based TBI studies, and the GOSE has been established as a “core” element of the National Institute of Neurological Disorders and Stroke (NINDS) TBI Common Data Elements (CDEs).^4^ However, the scale itself is not brain-specific and it does not distinguish what is caused by injury to the brain from disability caused by extracranial injury, which often occurs as part of the same incident. Pettigrew et al.^5^ state that, depending on the purpose for which the scale is used, it may be important to distinguish the effects of extracranial injuries from those caused specifically by the brain injury. The NINDS guidelines for the GOS and GOSE accurately describe the purpose of the measures as assessments of functioning and their validity as global outcome measures, but the CDEs lack any further instruction on how the forms should be used to assess disability excluding extracranial injuries thus emphasizing the effects of the brain injury versus all disability including extracranial injuries related to the index event. Most authors presenting GOSE data do not specify whether brain-injury-specific disability or disability due to the overall injury is being reported. Soon after Transforming Research and Clinical Knowledge in TBI (TRACK-TBI) was launched in 2014, TRACK-TBI investigators met with leaders of the Collaborative European NeuroTrauma Effectiveness Research in TBI (CENTER-TBI) study, a similar prospective observational study funded by the European Union. Both studies were using the GOSE as a primary outcome. This meeting revealed that, when administering the GOSE, TRACK-TBI was asking about limitations attributed to the TBI only and CENTER-TBI was asking about limitations attributed to injury to any part of the body. To harmonize these approaches, TRACK-TBI investigators modified their data collection procedure to evaluate both TBI-related disability (GOSE-TBI) and all injury-related disability (GOSE-All). The modified interview is included in the supplemental electronic material; it is also available on the Federal Interagency TBI Research (FITBIR) Informatics System,^6^ documenting a unique data element and on the TRACK-TBI website.^7^

The contribution of extracranial injuries to functional disability rating following TBI has received limited investigation. Dacey^8^ took the first systematic approach to determining the differential effects of head versus other system injuries on neuropsychological and psychosocial outcomes after injury. The authors found that at 1-month post-injury in their sample of people admitted to the hospital with TBI, extracranial injuries had a major impact on the ability of patients to return to work and to engage in day-to-day activities but not on traditional cognitive tasks. Participants’ ability to think, problem solve, or remember were affected only by the severity of brain injury, and not by extracranial injuries. However, everyday tasks were affected by both the severity of TBI and other injuries—and these everyday tasks (work, leisure activities, and travel) provide the basis of the scoring of the GOSE. Another study conducted in a single level 1 trauma center found that about half of patients with Glasgow Coma Scale (GCS) 13–15 TBI had been more bothered by their extracranial injury symptoms than their brain injury symptoms at 3 months post-injury.^9^ Prior studies have not assessed the extent to which estimates of disability following TBI are impacted by whether the instructions indicate all injury-related limitations (including those due to extracranial injuries) or only limitations attributed to the brain injury should be considered.

The current study aims to determine the extent to which GOSE scores that take into account the combined effect of the brain and extracranial injuries (GOSE-All scores) differ from scores excluding effects attributed to extracranial injuries (GOSE-TBI scores) among patients who were treated at Level I trauma centers in the United States. We characterized the effects of time since injury, TBI severity, and severity of extracranial injuries as factors influencing distributions of GOSE scores. We also examine the convergent and discriminant validity of the two scores. We hypothesized that discrepancies between GOSE-TBI and GOSE-All scores would be (1) greater at shorter rather than longer intervals post-injury, (2) greater for people experiencing less severe TBIs, and (3) greater for those with serious extracranial injuries. We also hypothesized that, compared with GOSE-All, GOSE-TBI would be more closely related to measures related to brain injury severity and less closely related to extracranial injury severity.

## Materials and Methods

### Participants

The TRACK-TBI study enrolled people as TBI participants who came to one of the 18 U.S. Level I trauma centers reporting at least a disturbance of consciousness due to physical trauma and who were ordered a head computed tomography (CT) scan as part of their clinical workup. Full inclusion and exclusion criteria can be found at the study website.^7^ This analysis included 2288 participants 17 years of age and older who were enrolled in the original TRACK-TBI U01 grant or its post-U01 extensions between February 26, 2014, and June 1, 2021, who had valid GOSE scores reflecting both disability due to all injuries and to the TBI, and who had known TBI and extracranial injury severity. Outcome was assessed at 2 weeks, 3 months, 6 months, and 12 months after injury in person or by phone.

### Measures

Demographic variables examined were age at injury, education, and gender. Education was assessed as the highest education level attained at the time of injury.

TBI severity was examined in three groups: GCS,^10^ score 13–15, CT− (mild, CT−) if post-resuscitation GCS score in the ED was 13–15 and the CT scan showed no acute intracranial abnormalities; GCS 13–15, CT+ (mild, CT+) if GCS was 13–15 and CT showed acute intracranial findings; GCS 3–12 (moderate/severe) if post-resuscitation GCS was 3–12 regardless of CT findings. Patients who were pharmacologically paralyzed were excluded. GCS was imputed for subjects who were intubated but had motor and eye components assessed.^11^

Extracranial injuries were assessed using the Abbreviated Injury Scale (AIS)^12,13^ for regions below the neck. Participants whose worst injury in these regions had a score of 3 or greater were classified as having a serious extracranial injury. Examples of serious extracranial injury include displaced long bone fracture or liver laceration. Participants discharged from the emergency department did not have AIS scores recorded and were included in the group with no serious extracranial injuries.

The GOSE structured interview was administered to patients who were able to provide responses or else to a family member or other person who knew the patient well before and after the injury. Respondents were first asked to indicate what extracranial injuries were sustained at the time of the index TBI. For each GOSE domain, respondents were then asked to rate the functional limitations due to the overall injury, as well as whether extracranial injuries contributed to the limitations in that area. If extracranial injuries contributed, respondents were then asked hypothetically what the functional limitations in that area would be if they did not have the extracranial injuries. The answers that included all injury-related limitations were used to derive the GOSE-All score while those that excluded extracranial injuries were used to calculate the GOSE-TBI score. Given this approach, injury-related post-traumatic stress symptoms contributed to both the GOSE-ALL and GOSE-TBI scores, as did other psychological and non-specific effects such as fatigue unless the participant attributed them solely to the extracranial injuries. When respondents indicated that extracranial injuries contributed, they were given the full range of responses for that domain to assess limitations excluding the extracranial injury; for example, if someone said they were unable to work and that was related to extracranial injuries, they could indicate that, excluding extracranial injuries, their work limitations were anywhere from unable to work to able to work to prior capacity.

Examiners were trained in administering the modified instrument and completed a taped interview with a mock respondent that was reviewed by an experienced member of the TRACK-TBI Outcomes Core, as described previously.^14^ In the training, if a respondent was not sure if a limitation such as relationship problems related to irritability or depression was caused solely by the extracranial injury, interviewers were encouraged to include the effect in the TBI rating. Generally, the study considered mental health and neurological issues that emerged or worsened after injury to contribute to both GOSE-All and GOSE-TBI scores unless individual circumstances indicated otherwise. A limitation related secondarily to the brain injury, such as limitations because of a bone flap that had not yet been replaced, was also to be attributed to the TBI. Respondents were left to decide how the effects of complications such as ventilator-associated pneumonia were reflected in their answers. Respondents were asked to discount the effects of new injuries not thought to be related to the index injury event for GOSE-TBI, but those effects were included in the GOSE-All score. Interviewers entered question-level response codes and notes to justify their ratings into the study database, which was reviewed by an experienced examiner for curation.^14^ Comparing the site examiner rating and the curated ratings, kappas ranged from 0.85 to 0.97 for GOSE-TBI and from 0.83 to 0.97 for GOSE-All, all in the range considered to be almost perfect.^15^ GOSE-TBI and GOSE-All scores range from 1 (death) to 8 (no injury-related limitations) and reflect the worst limitation across the domains. Except for participants who had died or were in a vegetative state, all domains were asked, even though the score had been determined by a domain earlier in the interview. GOSE data were centrally curated by experienced members of the TRACK-TBI Outcomes Core.^14^

### Data analysis

The percentage of patients with each GOSE score reflecting disability due to their brain injury (GOSE-TBI) as well as disability due to all aspects of their injury (GOSE-All) are shown using stacked bar graphs. Although GOSE-TBI and GOSE-All rate deaths and vegetative states the same because of the way the interview is structured, we included those scores in the analyses so the effect of each assessment method can be seen for the entire acute population entered; this decreased the apparent difference between the methods, especially for the GCS 3–12 subgroup. We calculated confidence intervals on the mean difference using a normal approximation based on the within-person difference between the two scores. Line plots portrayed the mean GOSE-TBI and GOSE-All scores as a function of time and extent of extracranial injury. To examine whether these differences depend on time, severity, and extent of extracranial injuries, we used a mixed effects linear model including time, TBI severity, and degree of extracranial injury as categorical fixed effects with participant effect modeled as a random intercept. An unstructured covariance matrix was assumed. Interactions were examined and were removed from the models if they were not significant. To explore the time effect within TBI severity and extracranial injury severity subgroups, linear mixed models were run in each subgroup. As a sensitivity analysis, a similar model was run on the ranks of the differences. We calculated Spearman correlations with measures of TBI and extracranial injury severity and compared the strength of correlations using a test of equality of dependent correlations.^16^ Analyses were carried out using SAS version 9.4.^17^

## Results

**FIG. 1.**
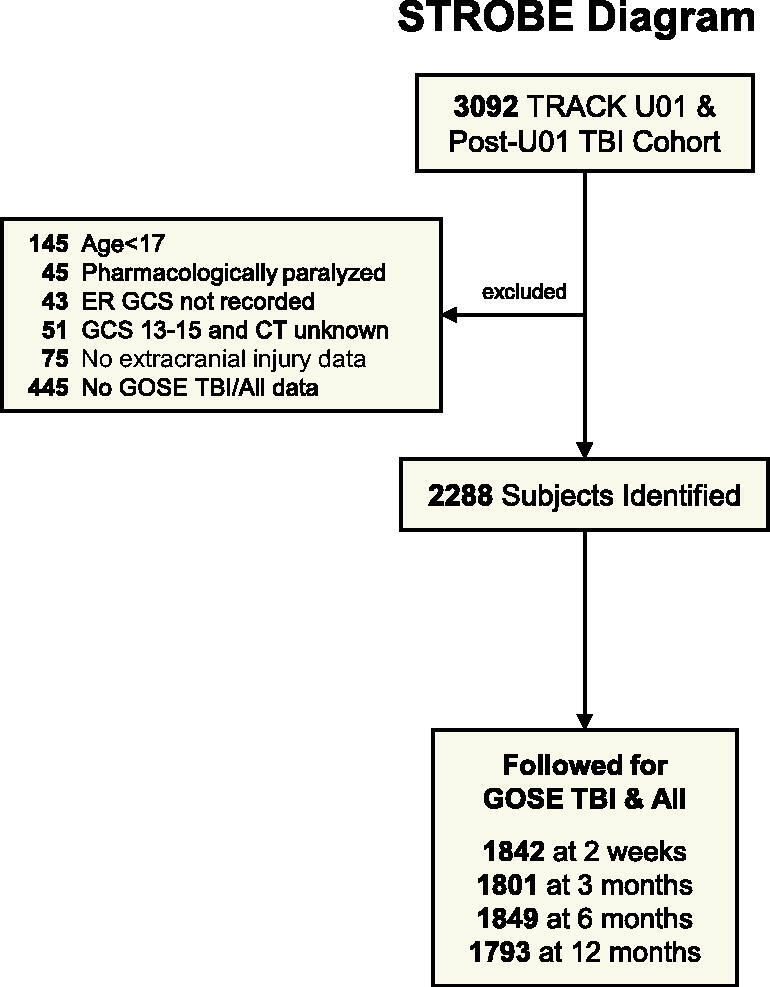
Participant inclusion (STROBE) diagram.

A STrengthening the Reporting of OBservational studies in Epidemiology (STROBE) diagram indicating participants included in the analysis is presented in Figure 1. Table 1 shows the demographics and injury characteristics of the included cases subdivided by TBI severity. The 2288 participants were primarily male (1569/2288 = 69%) with an average age of 42.3 years (SD = 17.8). Twenty-four percent (557/2288) had moderate/severe TBI, 30% (677/2288) had mild, CT+ TBI, 46% (1054/2288) had mild, CT− TBI and 20% (464/2288) had serious extracranial injuries. Comparing included and excluded adult (age 17 or older) cases (Supplementary Table S1), included cases were more likely to have a more severe TBI, have been enrolled in the TRACK-TBI U01 sample, be more highly educated, be discharged from the emergency department, and have no serious extracranial injury.

**Table 1.** Sample Descriptives

	Total	GCS 13–15 (Mild) CT−	GCS 13–15 (Mild) CT+	GCS 3-12 (Moderate/Severe)
Subjects	2288	1054	677	557
Source				
U01	2017 (88%)	966 (92%)	612 (90%)	439 (79%)
Post-U01	271 (12%)	88 (8%)	65 (10%)	118 (21%)
Age				
Mean (SD)	42.3 (17.8)	38.4 (15.7)	49.1 (19.1)	41.5 (17.6)
Sex				
Male	1569 (69%)	676 (64%)	468 (69%)	425 (76%)
Female	719 (31%)	378 (36%)	209 (31%)	132 (24%)
Education				
1-Less than HS	222 (10%)	98 (9%)	57 (9%)	67 (14%)
2-GED	120 (6%)	51 (5%)	35 (5%)	34 (7%)
3-High School only	1190 (55%)	566 (55%)	330 (51%)	294 (61%)
4-Undergrad degree	405 (19%)	203 (20%)	138 (21%)	64 (13%)
5-Graduate degree	228 (11%)	115 (11%)	87 (13%)	26 (5%)
Unknown	123	21	30	72
GCS ED				
Mean (SD)	12.6 (4.1)	14.8 (0.5)	14.6 (0.7)	5.9 (3.1)
CT positive				
No	1093 (49%)	1054 (100%)	—	39 (8%)
Yes	1158 (51%)	—	677 (100%)	481 (93%)
Unknown	37	0	0	37
Admitted				
No	485 (21%)	431 (41%)	50 (7%)	4 (1%)
Yes	1803 (79%)	623 (59%)	627 (93%)	553 (99%)
Serious extracranial injury				
No (Worst extracranial AIS <3)	1824 (80%)	875 (83%)	579 (86%)	370 (66%)
Yes (Worst extracranial AIS ≥3)	464 (20%)	179 (17%)	98 (14%)	187 (34%)
GOSE counts				
2–4 weeks TBI + All	1842 (81%)	794 (75%)	564 (83%)	484 (87%)
3 months TBI + All	1801 (79%)	815 (77%)	527 (78%)	459 (82%)
6 months TBI + All	1849 (81%)	860 (82%)	539 (80%)	450 (81%)
12 months TBI + All	1793 (78%)	815 (77%)	545 (81%)	433 (78%)

AIS, Abbreviated Injury Scale; CT, computed tomography; GCS, Glasgow Coma Scale; TBI, traumatic brain injury; SD, standard deviation; HS, High School; GED, General Educational Development test; ED, Emergency Department; GOSE, Glasgow Outcome Scale Extended.

**FIG. 2.**
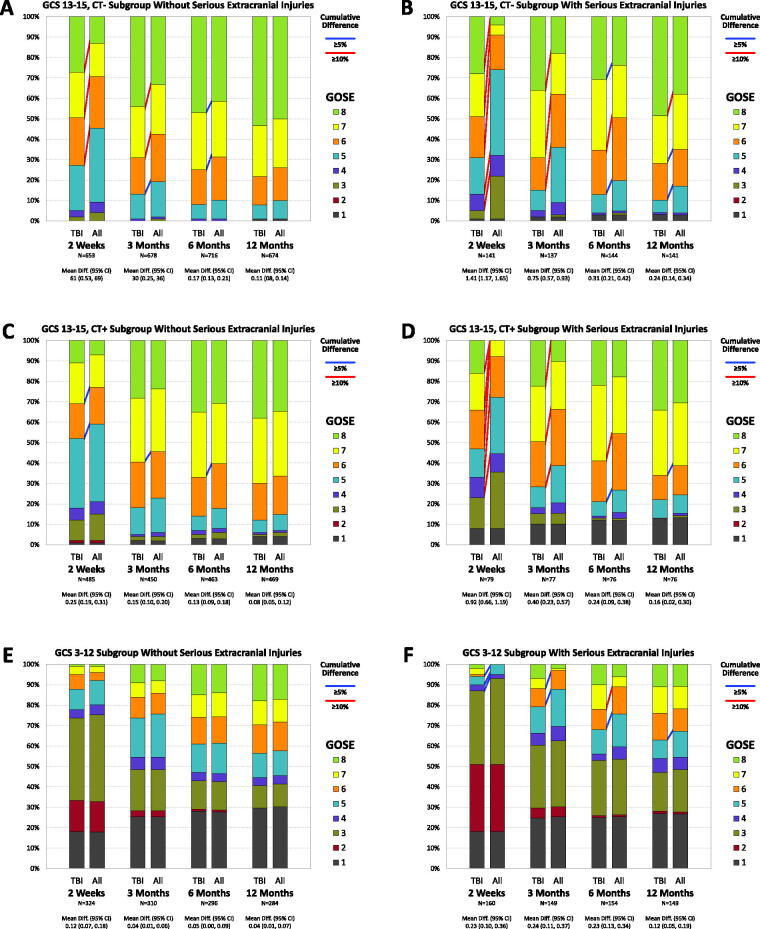
Stacked bar charts of GOSE-TBI and GOSE-All by time since injury, TBI severity, and serious extracranial injuries. Blue lines connect cumulative percents with 5 but under 10 percentage point discrepancy and red lines connect those with at least 10 percentage point discrepancy. **(A)** GCS 13–15, CT− subgroup without serious extracranial injuries. **(B)** GCS 13–15, CT− subgroup with serious extracranial injuries. **(C)** GCS 13–15, CT+ subgroup without serious extracranial injuries. **(D)** GCS 13–15, CT+ subgroup with serious extracranial injuries. **(E)** GCS 3–12 subgroup without serious extracranial injuries. **(F)** GCS 3–12 subgroup with serious extracranial injuries. GCS, Glasgow Coma Scale; GOSE, Glasgow Outcome Scale Extended; TBI, traumatic brain injury.

Figure 2 shows the percent with each GOSE-All and GOSE-TBI score, reflected as the height of each individual color. The top of each color bar shows the percent with unfavorable outcomes if unfavorable outcomes were defined as the GOSE scores dichotomized at that color bar. The percentages with unfavorable outcomes are connected between TBI and All bars where the difference between bars is 5–9 (blue) or ≥10 (red) percentage points. The extent of difference depends on each time, TBI severity, and extracranial injury severity, with a quantitative 3-way interaction (*p* < 0.001, Supplementary Table S1) and consistent patterns. Similar stacked bars collapsed over the extent of extracranial injury are provided in Supplementary Figure S1.

Figure 2A and B displays scores for the GCS 13–15 CT− subgroup without (2A) and with (2B) serious extracranial injuries. At 2 weeks, there is a 42 percentage point difference in the proportion of participants with lower moderate disability (GOSE 5) or worse in those with serious extracranial injuries (33% on GOSE-TBI compared with 75% on GOSE-All). The biggest difference among those without serious extracranial injuries is 21 percentage points in the proportion with upper moderate recovery (GOSE 6) or worse at 2 weeks. These differences decrease with time in both subgroups, with the largest differences of 4 percentage points (for proportion with upper moderate recovery GOSE 6 or worse) and 11 percentage points (for proportion with lower good recovery GOSE 7 or worse) in those without and with serious extracranial injuries by 12 months.

Figure 2C and D displays the scores for those with GCS 13–15, CT+ without (2C) and with (2D) serious extracranial injuries. The largest discrepancies are 9 percentage points in those without serious extracranial injuries (2C) and 29 percentage points in those with serious extracranial injuries (both at 2 weeks in the proportion with upper moderate recovery GOSE 6 or worse). By 12 months, the largest discrepancies are 3 and 6 percentage points.

Figure 2E and F displays the scores for those with GCS 3–12 without (2E) and with (2F) serious extracranial injuries. In those without serious extracranial injuries, the largest discrepancy goes from 5 percentage points at 2 weeks to 1 percentage point at 12 months. In those with serious extracranial injuries, the worst difference is in the 5–10 percentage point range with no particular pattern with time.

At all times and in both extracranial injury severity groups, Figure 2 shows the largest discrepancies in those with GCS 13–15, CT-TBIs with decreasing differences with increasing TBI severity. Those with serious extracranial injury show greater differences for each TBI severity and time. In all but the GCS 3–12 TBI group with serious extracranial injuries, differences decrease with time.

**FIG. 3.**
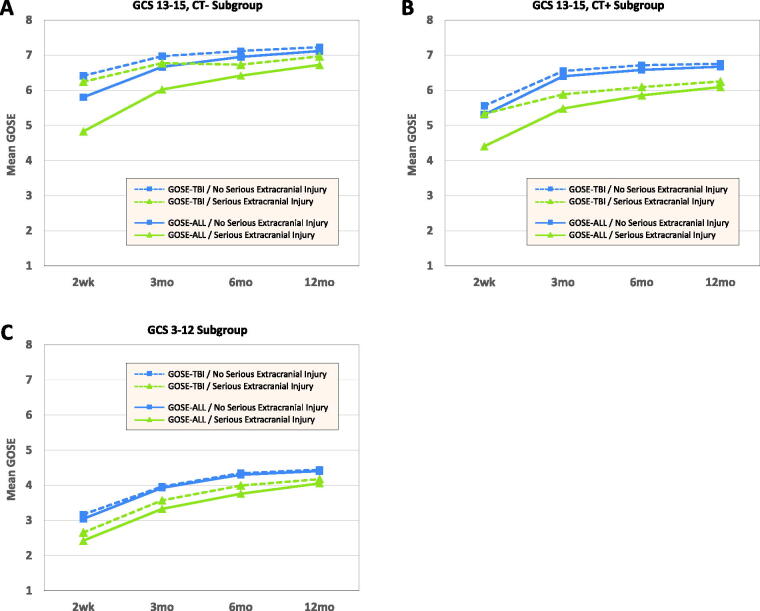
Mean GOSE-TBI and GOSE-All by time since injury and serious extracranial injuries. **(A)** GCS 13–15, CT− subgroup. **(B)** GCS 13–15, CT+ subgroup. **(C)** GCS 3–12 subgroup. CT, computer tomography; GCS, Glasgow Coma Scale; GOSE, Glasgow Outcome Scale Extended; TBI, traumatic brain injury.

Figure 3 depicts the mean GOSE-TBI and GOSE-All scores in line plots. The means show similar patterns to the maximum individual score discrepancies with the difference decreasing with more severe TBI and a longer time since the injury and a bigger difference in participants with serious extracranial injuries.

A mixed effects model examines the statistical significance of the patterns (Supplementary Table S2). The modeled mean difference in each subgroup at each time is shown in Supplementary Table S3. The modeled mean difference is the estimate from the mixed effects model of the average GOSE-TBI minus GOSE-All in the indicated subgroup at the given assessment. Each effect, including the 3-way interaction of TBI severity, extracranial injury severity, and time, is significant (each *p* < 0.001). The patterns of significant effects are consistent with differences decreasing with worsening TBI severity, later times after injury, and no serious extracranial injuries. The non-significant comparisons suggest that the effect of serious extracranial injury is similar in the GCS 13–15 groups with and without CT abnormalities and that there is little effect of time in those with GCS 3–12 and serious extracranial injuries. A similar analysis done on the ranks of the difference to assess sensitivity to non-normality shows a similar pattern of significance.

Correlations of GOSE-TBI and GOSE-All with measures of TBI and extracranial injury severity are shown in Table 2. All correlations except with AIS-extracranial were in the range of 0.35–0.62 (each *p* < 0.001), The correlations of GOSE-TBI and GOSE-All with GCS score were almost identical. GOSE-TBI was more closely correlated than GOSE-All with the AIS-head at all times and with the TBI severity group at all but 6 months. AIS-extracranial was more highly correlated with GOSE-All than GOSE-TBI at all times (each *p* < 0.001).

**Table 2.** Spearman Correlation of GOSE-All and GOSE-TBI with Measures of Brain Injury Severity and Non-Brain Injury Severity

Correlations	2 weeks			3 months			6 months			12 months		
	GOSE-All	GOSE-TBI	*p* ^a^	GOSE-All	GOSE-TBI	*p* ^a^	GOSE-All	GOSE-TBI	*p* ^a^	GOSE-All	GOSE-TBI	*p* ^a^
GCS score	0.58	0.59	0.147	0.48	0.49	0.170	0.42	0.41	0.225	0.41	0.42	0.087
TBI severity^b^	0.56	0.62	<0.001	0.47	0.51	<0.001	0.42	0.43	0.130	0.42	0.44	0.003
AIS-head^c^	−0.46	−0.53	<0.001	−0.40	−0.47	<0.001	−0.37	−0.39	0.024	−0.35	−0.37	0.002
AIS-extracranial^c^	−0.25	−0.07	<0.001	−0.22	−0.09	<0.001	−0.15	−0.09	<0.001	−0.09	−0.06	<0.001

^a^ *p*-Value from the test of equality of the correlation with GOSE-All and GOSE-TBI.

^b^ TBI severity GCS 3–8, GCS 9–12, GCS 13–15 CT+, GCS 13–15 CT− coded 1, 2, 3, 4.

^c^ Hospitalized cases only.

AIS, Abbreviated Injury Scale; CT, computed tomography; GCS, Glasgow Coma Scale; TBI, traumatic brain injury.

## Discussion

Including limitations attributed to non-brain injury when evaluating the GOSE can change the level of functional limitation reported by people who have sustained a TBI. The impact of this decision varies by time since injury and TBI subpopulation. The average GOSE score differs by a full point or more when GOSE is evaluated a few weeks after a mild TBI with serious extracranial injuries to about a 20th of a point in those with moderate or severe TBI without serious extracranial injuries evaluated months after their injuries. The findings supported all three hypotheses—that the extent of the difference is greater with milder TBIs, with serious extracranial injuries, and soon after the injury. GOSE-TBI and GOSE-All are each moderately correlated to measures of brain injury severity, with GOSE-TBI being more strongly related than GOSE-All on some, but not all measures.

Neither approach to scoring the GOSE is right or wrong. Which approach is preferable depends on the purpose of the study. If one is evaluating a relationship of brain-injury-specific biomarkers to the outcome or looking for an outcome for a clinical trial of a treatment that affects only the brain, GOSE-TBI may be more sensitive. If, however, one is interested in describing the overall impact of an injury on a person’s functioning or one wants to balance the positive effects on the brain of a potential treatment with adverse side effects on the cardiac or respiratory system, GOSE-All might provide that balance.

The understanding that there are different possibilities for including extracranial injuries when assessing disabilities using GOSE allows investigators to emphasize what is intended in explaining the measure to the respondent. Although the seminal article on the GOSE structured interview^5^ mentions that the interview can be used to collect disability related solely to the TBI or more broadly, the interview form provided and subsequently how the GOSE is described on the CDE website,^4^ is such that either approach examined in this article can be taken. Unless the instructions clearly state what causes of functional limitation are to be included, each study respondent and each outcome examiner will interpret the interview questions through their own lens, increasing variability and decreasing interpretability of the findings.

The NINDS TBI CDEs were created to help researchers in planning studies and to assist in communications and data sharing. Those roles are compromised by the silence of the CDEs about how extracranial injuries are dealt with. The CDEs for TBI should be clarified to indicate that there are multiple approaches that can be targeted with a functional outcome measure such as GOSE and information should be collected so users of the data, including investigators obtaining it from data repositories such as FITBIR, can easily know what scoring approach has been used in a given study.

Whether using the standard interview in the Wilson et al.^5^ article introduced to the interviewee to indicate that effects of extracranial injuries are to be discounted or the interview developed by the TRACK-TBI investigators to determine both GOSE-All and GOSE-TBI (Supplementary Data), assessment of GOSE-TBI is not based solely on observable changes in functioning. It requires the respondent to assess whether the participant would have limitations with activities they have not attempted because of extracranial injuries. It also requires them to decide whether the extracranial injuries are the sole cause of functional limitations that can be attributable to either brain or extracranial injury, such as fatigue, mental health issues, or balance problems. The guidance of a skilled outcome examiner can help the respondent in making these decisions but the inherent difficulties may lead GOSE-TBI to either under- or over-estimate the effects of the TBI, increasing variability and potentially inducing bias. Notwithstanding these challenges, we have found GOSE-TBI to be robustly associated with objective TBI biomarkers,^18,19^ supporting its validity for certain indications. The equivalent correlation of GOSE-TBI and GOSE-All with GCS and the stronger correlation of GOSE-TBI with ordinal TBI severity and AIS-head in the first 3 months after injury adds to the evidence for the validity of GOSE-TBI, despite the difficulties inherent in parsing out the specific effects of extracranial injuries. The stronger association of GOSE-All with AIS-extracranial confirms discriminant validity. An alternative approach to accounting for extracranial severity is to adjust for the maximum AIS-extracranial score in the analysis. We looked at such an adjustment for patients who were hospitalized and the correlations of this adjusted score with measures associated with TBI severity did not increase the correlation over that of GOSE-All by more than 0.01 (data not shown). Future work should include an assessment of the inter-rater reliability of the GOSE-TBI and GOSE-All and of the relative strength of their relationship with imaging and blood-based biomarkers.

Study limitations include that TRACK-TBI enrolled a convenience sample of patients coming to Level I trauma centers and receiving an order for a head CT as part of their clinical care. The study therefore is not representative of all people with TBI. Another limitation relates to the classification of extracranial injuries. Participants discharged from the ED had no information recorded about the extent of their extracranial injuries. They were assumed to be at worst moderate, i.e., maximum extracranial AIS 2 or less. Those admitted to the hospital had the AIS scores collected, but these scores were developed to relate to mortality risk, not disability risk, and thus are not an ideal measure to account for functional limitations after an injury. Dichotomizing these scores might further limit their usefulness.

## Conclusions

The inclusion of the effects of extracranial injuries can impact the GOSE score distribution substantially. Differences are largest at early follow-ups, for people with less severe TBI, and for people with serious extracranial injuries. Researchers should thoughtfully decide which GOSE assessment method is best suited to answer their questions and authors should report which method was used. GOSE CDEs should indicate that the GOSE can be collected in several ways and provide a qualifier to indicate what method was used. Data repositories should provide easy ways for data users to determine what assessment method was used.

## Transparency, Rigor, and Reproducibility

The TRACK-TBI study was pre-registered at clinicaltrials.gov (clinicaltrials.gov NCT2119182). All data used for this investigation are available by request from FITBIR (https://fitbir.nih.gov/). All codes and scripts used for this investigation are available from the corresponding author upon reasonable request. The analysis plan was not formally preregistered. The sample size of 2288 was based on the number of cases with available data. The TRACK-TBI clinical protocol and outcome procedures are available at https://tracktbi.ucsf.edu/researchers. The analysis plan was not formally preregistered. Statistical tests used were based on the assumption of normality but are robust with large sample sizes such as those available here. Non-independence of measurements has been addressed using mixed effects models, as reported in the text. Data curation was performed by the TRACK-TBI Outcomes Core. The key inclusion criteria and outcome measures are standards in the field. Key inclusion criteria and clinical outcomes were assessed by trained study staff with oversight by clinicians and investigators with extensive experience in the assessment of persons with TBI. Statistical analyses were performed using SAS, version 9.4. Statistical analysis was performed by J.B., with oversight by N.T., who co-leads the TRACK-TBI Biostatistical and Comparative Effectiveness Research Core. No replication or external validation studies have been performed or are planned/ongoing at this time to our knowledge. The authors agree to publish the article using the Mary Ann Liebert Inc. “Open Access” option under the appropriate license.

## The TRACK-TBI Investigators

Neeraj Badjatia (University of Maryland); Shawn Eagle (University of Pittsburgh); Shankar Gopinath (Baylor College of Medicine); Ramesh Grandhi (University of Utah); Vijay Krishnamoorthy (Duke University); Christine Mac Donald (University Washington); Michael McCrea (Medical College of Wisconsin); Randall Merchant (Virginia Commonwealth University); Pratik Mukherjee (University of California, San Francisco); Laura B. Ngwenya (University of Cincinnati); David Okonkwo (University of Pittsburgh); Claudia Robertson (Baylor College of Medicine); Richard B Rodgers (Goodman Campbell Brain and Spine); David Schnyer (UT Austin); Sabrina R. Taylor (University of California, San Francisco); Mary Vassar (University of California, San Francisco); John K. Yue (University of California, San Francisco); Ross Zafonte (Harvard Medical School).
