# Supplementary material for: Contribution of Extracranial Injuries to GOSE Scores after Traumatic Brain Injury TBI: A TRACK-Traumatic Brain Injury Study
Source: J Neurotrauma. 2025 Sep 12;42(17-18):1550–9. doi: 10.1089/neu.2024.0421 (PMC12677206; doi:10.1089/neu.2024.0421)
Supplement: Supplementary file 1 [file neu.2024.0421_supp_data.pdf]

DATE ADMINISTERED__________ SUBJECT ID_____ START TIME______

# GLASGOW OUTCOME SCALE – EXTENDED REVISED (GOSE-Revised)

The GOSE-Revised was created in an attempt to document the impact peripheral or non-CNS injuries obtained at the time of the study related injury have on the final rating. Two scores will be obtained. The first will be an 'All' rating which looks at the participant's change in overall level of dependence as a function of the injury. This score will include effects of the TBI (brain) as well as peripheral injuries sustained in the same accident. The second score will remove the obvious impact of the peripheral injuries leaving the disability rating that may be due to the TBI.* Note that on any given section below, the TBI rating is the same as the All rating if peripheral injuries do not contribute to the limitations there (determined by questions 2d, 3c, 4c, 5d, 6d, 7d or 8c).
(*If a new injury occurs after the study injury, include the *cumulative effects of all brain and peripheral injuries* in the 'All' rating. The 'TBI' score will remove the effects of *all* peripheral injuries,

The same format will be applied to each set of questions. For example, the GOSE-Revised would be administered as follows for travel outside the home.

Assess any limitations with travel as always for questions 4a and 4b. If travel is limited ask,
4c. '*Does your _____________ (broken leg) contribute to the limitations with travel?'*
*4d. Then, 'If you did not have the _______________ (broken leg) do you think you would be able to travel locally without assistance?'*
If travel is not limited proceed to question 5a skipping 4c and 4d.

| | | |
|---|---|---|
| **PERIPHERAL INJURIES**<br>A. Did you sustain any other system injuries or peripheral injuries (e.g., fractured limbs, spinal cord injury, complications from other system surgeries, etc.) in addition to your TBI? | 1 ☐ No | 2☐ Yes (record all of these injuries on the lines provided) |
| A1. Record all peripheral type injuries on the lines provided and refer to them specifically below as noted*:<br>(*If a new injury occurs after the study injury, record all peripheral injuries below)<br>______________________________<br>______________________________<br>______________________________<br>______________________________<br>______________________________<br>______________________________ | | |

DATE ADMINISTERED__________ SUBJECT ID__________

| **CONSCIOUSNESS** | | |
|---|---|---|
| 1. Is the head injured person able to obey simple commands or say any words? | 1☐ No (VS) | 2 ☐ Yes |
| *Anyone who shows ability to obey even simple commands or utter any word or communicate specifically in any other way is no longer considered to be in the vegetative state. The examiner should review the results of the CRS-R and consult with nursing/clinical staff before assigning a rating of vegetative state on question #.1* | | |

| **INDEPENDENCE IN THE HOME** | | |
|---|---|---|
| 2a. Is the assistance of another person at home essential every day for some activities of daily living?<br>*For a 'No' answer they should be able to look after themselves at home for 24 hours if necessary, though they need not actually look after themselves. Independence includes the ability to plan for and carry out the following activities: getting washed, putting on clean clothes without prompting, preparing food for themselves, dealing with callers, and handling minor domestic crises. The person should be able to carry out activities without needing prompting or reminding, and should be capable of being left alone overnight.* | 1☐ No-**go to 3a** | 2☐ Yes-**go to 2b** |
| 2b. Do you need frequent help or someone to be around at home most of the time?<br>*For a 'No' answer they should be able to look after themselves at home for up to 8 hours during the day if necessary, though they need not actually look after themselves.* | 1 ☐ No (Upper SD All) | 2 ☐ Yes (Lower SD All) |
| 2c. Was assistance at home essential before the injury? | 1 ☐ No | 2 ☐ Yes |
| 2d. Does your ______________________ (specify problem(s) identified in question "A" above – e.g., your broken leg) contribute to the limitations described here? | 1 ☐ No-**go to 3a** (TBI rating = All rating) | 2 ☐ Yes-**go to 2e** |
| 2e. If you did not have ________________________ (specify problem(s) identified in question "A" above), do you think the assistance of another person at home would be essential every day for some activities of daily living?<br>*For a 'No' answer they should be able to look after themselves at home for 24 hours if necessary, though they need not actually look after themselves. Independence includes the ability to plan for and carry out the following activities: getting washed, putting on clean clothes without prompting, preparing food for themselves, dealing with callers, and handling minor domestic crises. The person should be able to carry out activities without needing prompting or reminding, and should be capable of being left alone overnight.* | 1 ☐ No-**go to 3a** | 2 ☐ Yes-**go to 2f** |
| 2f. If you did not have ________________________ (specify problem(s) identified in question "A" above), would you need frequent help or someone to be around at home most of the time?<br>*For a 'No' answer they should be able to look after themselves at home for up to 8 hours during the day if necessary, though they need not actually look after themselves* | 1 ☐ No (Upper SD TBI) | 2 ☐ Yes (Lower SD TBI) |

| **INDEPENDENCE OUTSIDE THE HOME** | | |
|---|---|---|
| 3a. Are you able to shop without assistance?<br>*This includes being able to plan what to buy, take care of money themselves, and behave appropriately in public. They need not normally shop, but must be able to do so.* | 1 ☐ No (Upper SD all) | 2 ☐ Yes-**go to 4a** |
| 3b. Were you able to shop without assistance before the injury? | 1 ☐ No | 2 ☐ Yes |
| 3c. Does your ________________________ (specify problem(s) identified in question "A" above – e.g., your broken leg) contribute to limitations with shopping? | 1 ☐ No-**go to 4a** (TBI rating = All rating) | 2 ☐ Yes-**go to 3d** |
| 3d. If you did not have ________________________ (specify problem(s) identified in question "A" above), do you think you would be able to shop without assistance? | 1 ☐ No (Upper SD TBI) | 2 ☐ Yes |
| 4a. Are you able to travel locally without assistance?<br>*They may drive or use public transport to get around. Ability to use a taxi is sufficient, provided the person can phone for it themselves.* | 1 ☐ No (Upper SD All) | 2 ☐ Yes-**go to 5a** |
| 4b. Were you able to travel without assistance before the injury? | 1 ☐ No | 2 ☐ Yes |
| 4c. Does your ________________________ (specify problem(s) identified in question "A" above – e.g., your broken leg), contribute to limitations with travel? | 1 ☐ No-**go to 5a** (TBI rating = All rating) | 2 ☐ Yes-**go to 4d** |
| 4d. If you did not have ________________________ (specify problem(s) identified in question "A" above), do you think you would be able to travel locally without assistance? | 1 ☐ No (Upper SD TBI) | 2 ☐ Yes |

DATE ADMINISTERED__________ SUBJECT ID__________

| | | |
|---|---|---|
| **WORK**<br>5a. Were you either working or seeking employment before the injury (answer 'yes') or were you doing neither (answer 'no')?<br>(If not considered a worker, (i.e. retired, homemaker, permanently disabled) , mark 5A as "No" and 5B as "Yes") | 1 ☐ No | 2 ☐ Yes |
| 5b. Are you currently able to work to your previous capacity?<br>*If you were working before, then your current capacity for work should be at the same level. If you were seeking work before, then the injury should not have adversely affected your chances of obtaining work or the level of work for which you are eligible. If you were a student before the injury then your capacity for study should not have been adversely affected.* | 1 ☐ No-**go to 5c** | 2 ☐ Yes-**go to 6a** |
| 5c. How restricted are you?<br>1☐ Reduced work capacity (Upper MD All)<br>2☐ Able to work only in a sheltered workshop or non-competitive job or currently unable to work (Lower MD All) | | |
| 5d. Does your ________________________ (specify problem(s) identified in question "A" above – e.g., your broken leg) contribute to limitations with work/school? | 1 ☐ No-**go to 6a** (TBI rating = All rating) | 2 ☐ Yes-**go to 5e** |
| 5e. If you did not have _____________________ (specify problem(s) identified in question "A" above), do you think you would be able to work to your previous capacity? | 1 ☐ No-**go to 5f** | 2 ☐ Yes-**go to 6a** |
| 5f. If you did not have ________________________ (specify problem(s) identified in question "A" above), how restricted do you think you would be?<br>1☐ Reduced work capacity (Upper MD TBI)<br>2☐ Able to work only in a sheltered workshop or non-competitive job or currently unable to work (Lower MD TBI) | | |

| | | |
|---|---|---|
| **SOCIAL & LEISURE ACTIVITIES**<br>6a. Did you engage in regular social and leisure activities outside home before the injury? | 1 ☐ No | 2☐ Yes |
| 6b. Are you able to resume regular social and leisure activities outside the home? | 1 ☐ No-**go to 6c** | 2☐ Yes – **go to 7a** |
| 6c. What is the extent of restriction on your social and leisure activities?<br>1 ☐ Participate a bit less: at least half as often as before injury (Lower GR All)<br>2 ☐ Participate much less: less than half as often (Upper MD All)<br>3 ☐ Unable to participate: rarely, if ever, take part (Lower MD All) | | |
| 6d. Does your ________________________ (specify problem(s) identified in question "A" above – e.g., your broken leg) contribute to limitations with social and leisure activities? | 1 ☐ No-**go to 7a** (TBI rating = All rating) | 2☐ Yes-**go to 6e** |
| 6e. If you did not have ___________________ (specify problem(s) identified in question "A" above), do you think you would be able to resume regular social and leisure activities outside the home? | 1 ☐ No-**go to 6f** | 2 ☐ Yes – **go to 7a** |
| 6f. If you did not have ________________________ (specify problem(s) identified in question "A" above), how restricted do you think you would be?<br>1 ☐ Participate a bit less: at least half as often as before injury (Lower GR TBI)<br>2 ☐ Participate much less: less than half as often (Upper MD TBI)<br>3 ☐ Unable to participate: rarely, if ever, take part (Lower MD TBI) | | |

| | | |
|---|---|---|
| **FAMILY & FRIENDSHIPS**<br>7a. Have there been psychological problems which have resulted in ongoing family disruption or disruption to friendships?<br>*Typical post-traumatic personality changes: quick temper, irritability, anxiety, insensitivity to others, mood swings, depression, and unreasonable or childish behavior* | 1 ☐ No-**go to 8a** | 2☐ Yes-**go to 7b** |
| 7b. What has been the extent of disruption or strain?<br>1 ☐ Occasional – less than weekly (Lower GR All)<br>2 ☐ Frequent – once a week or more, but tolerable (Upper MD All)<br>3 ☐ Constant – daily and intolerable (Lower MD All) | | |

DATE ADMINISTERED__________ SUBJECT ID__________

| | | |
|---|---|---|
| 7c. Were there problems with family or friends before the injury?<br>*If there were some problems before injury, but these have become markedly worse since injury then answer 'no' to this question* | 1☐ No | 2☐ Yes |
| 7d. Does your _________________________ (specify problem(s) identified in question "A" above – e.g., your broken leg) contribute to limitations with family & friendships? | 1☐ No-**go to 8a** (TBI rating = All rating) | 2☐ Yes-**go to 7e** |
| 7e. If you did not have _______________________ (specify problem(s) identified in question "A" above), do you think there would be psychological problems which result in ongoing family disruption or disruption to friendships? | 1☐ No-**go to 8a** | 2☐ Yes-**go to 7f** |
| 7f. If you did not have _________________________ (specify problem(s) identified in question "A" above), what do you think the extent of the disruption or strain would be?<br>1 ☐ Occasional – less than weekly (Lower GR TBI)<br>2 ☐ Frequent – once a week or more, but tolerable (Upper MD TBI)<br>3 ☐ Constant – daily and intolerable (Lower MD BI) | | |

| | | |
|---|---|---|
| **<u>RETURN TO NORMAL LIFE</u>**<br>8a. Are there any other current problems relating to the injury which affect daily life?<br>*Other typical problems reported after head injury: headaches, dizziness, tiredness, sensitivity to noise or light, slowness, memory failures, and concentration problems* | 1☐ No (Upper GR All) – **End here** | 2☐ Yes (Lower GR All) |
| 8b. Were similar problems present before the injury?<br>*If there were some problems before injury, but these have become markedly worse since injury then answer 'no' to this question* | 1☐ No | 2☐ Yes |
| 8c. Does your _________________________ (specify problem(s) identified in question "A" above – e.g., your broken leg) contribute to any other current problems which affect daily life? | 1☐ No- **End here** (TBI rating = All rating) | 2☐ Yes-**go to 8d** |
| 8d. If you did not have _________________________ (specify problem(s) identified in question "A" above), do you think you would have current problems that affect daily life? | 1☐ No (Upper GR TBI) | 2☐ Yes (Lower GR TBI) |

DATE ADMINISTERED__________ SUBJECT ID__________

The patient's overall rating is based on the lowest outcome category indicated on the scale. Refer to guidelines for further information concerning administration and scoring. Record the lowest score due to all injury related limitations (GOSE-All) and the lowest score with any limitations from other system injuries removed (GOSE-TBI).

| | | |
|---|---|---|
| 1 | Dead | |
| 2 | Vegetative State (VS) | GOSE-All Score: ______ |
| 3 | Lower Severe Disability (Lower SD) | |
| 4 | Upper Severe Disability (Upper SD) | |
| 5 | Lower Moderate Disability (Lower MD) | GOSE-TBI Score: ______ |
| 6 | Upper Moderate Disability (Upper MD) | |
| 7 | Lower Good Recovery (Lower GR) | |
| 8 | Upper Good Recovery (Upper GR) | |

Describe the person's situation below and provide details about the reasons for each GOSE rating.

Test Completion Code (circle one):

/ 1.0 / 1.1 / 1.2 / 1.3 / 2.2 /2.3 / 2.4 / 2.5 / 2.6 / 3.2 / 3.3 / 3.4 / 3.5 / 3.6 / 4.0 / 5.0 /

If 1.2 or 5.0 (Other) Please Specify: ________________________________ END TIME______
