# Supplementary material for: Contribution of Extracranial Injuries to GOSE Scores after Traumatic Brain Injury TBI: A TRACK-Traumatic Brain Injury Study
Source: J Neurotrauma. 2025 Sep 12;42(17-18):1550–9. doi: 10.1089/neu.2024.0421 (PMC12677206; doi:10.1089/neu.2024.0421)
Supplement: Supplementary file 2 [file neu.2024.0421_supp_figures1.docx]

eFigure 1. Stacked bar charts of GOSE-TBI and GOSE-All by time since injury and TBI severity. Blue lines connect cumulative percents with 5 but under 10 percentage point discrepancy and red lines connect those with at least 10 percentage point discrepancy.
