# Supplementary material for: Contribution of Extracranial Injuries to GOSE Scores after Traumatic Brain Injury TBI: A TRACK-Traumatic Brain Injury Study
Source: J Neurotrauma. 2025 Sep 12;42(17-18):1550–9. doi: 10.1089/neu.2024.0421 (PMC12677206; doi:10.1089/neu.2024.0421)
Supplement: Supplementary file 3 [file neu.2024.0421_supp_tables1.docx]

**eTable 1- Sample Descriptives comparing included and excluded cases.**

|  | **Total** | **Included** | **Excluded** | **SMD** | **p** |
| --- | --- | --- | --- | --- | --- |
|  |  |  |  |  |  |
| **Subjects** | **2947** | **2288** | **659** |  |  |
|  |  |  |  |  |  |
| **Severity** |  |  |  |  |  |
| A - Mild CT- | 1348 (48%) | 1054 (46%) | 294 (57%) | -0.02 | ^<^.001 |
| B - Mild CT+ | 832 (30%) | 677 (30%) | 155 (30%) | -0.06 |  |
| C - Mod/Sev | 628 (22%) | 557 (24%) | 71 (14%) | -0.14 |  |
| Unknown | 139 | 0 | 139 | -0.21 |  |
|  |  |  |  |  |  |
| **Source** |  |  |  |  |  |
| U01 | 2552 (87%) | 2017 (88%) | 535 (81%) | 0.07 | ^<^.001 |
| Post-U01 | 395 (13%) | 271 (12%) | 124 (19%) | -0.07 |  |
|  |  |  |  |  |  |
| **Age** |  |  |  |  |  |
| Mean (SD) | 42.3 (17.8) | 42.3 (17.8) | 42.0 (17.6) | -0.03 | .686 |
|  |  |  |  |  |  |
| **Sex** |  |  |  |  |  |
| Male | 2034 (69%) | 1504 (69%) | 530 (70%) | 0.02 | .339 |
| Female | 913 (31%) | 683 (31%) | 230 (30%) | -0.02 |  |
|  |  |  |  |  |  |
| **Education** |  |  |  |  |  |
| 1 - Less than HS | 339 (12%) | 222 (10%) | 117 (20%) | -0.08 | ^<^.001 |
| 2 – GED | 155 (6%) | 120 (6%) | 35 (6%) | 0.00 |  |
| 3 - High School only | 1505 (55%) | 1190 (55%) | 315 (55%) | -0.04 |  |
| 4 - Undergrad degree | 469 (17%) | 405 (19%) | 64 (11%) | -0.08 |  |
| 5 - Graduate degree | 268 (10%) | 228 (11%) | 40 (7%) | -0.04 |  |
| Unknown | 211 | 123 | 88 | -0.08 |  |
|  |  |  |  |  |  |
| **GCS ED** |  |  |  |  |  |
| Mean (SD) | 12.8 (4.0) | 12.6 (4.1) | 13.6 (3.2) | 0.38 | ^<^.001 |
| Unknown | 88 | 0 | 88 | 0.36 |  |
|  |  |  |  |  |  |
| **CT Positive** |  |  |  |  |  |
| No | 1420 (50%) | 1093 (49%) | 327 (56%) | 0.08 | .001 |
| Yes/Suspected | 1414 (50%) | 1158 (51%) | 256 (44%) | -0.08 |  |
| Unknown | 113 | 37 | 76 | -0.10 |  |
|  |  |  |  |  |  |
| **Admitted** |  |  |  |  |  |
| No | 582 (20%) | 485 (21%) | 97 (15%) | 0.07 | ^<^.001 |
| Yes | 2365 (80%) | 1803 (79%) | 562 (85%) | -0.07 |  |
|  |  |  |  |  |  |
| **Serious Extracranial Injury** |  |  |  |  |  |
| No (Worst Extracranial AIS<3) | 2256 (79%) | 1824 (80%) | 432 (76%) | 0.04 | .039 |
| Yes (Worst Extracranial AIS≥3) | 603 (21%) | 464 (20%) | 139 (24%) | -0.04 |  |
| Unknown | 88 | 0 | 88 | -0.13 |  |
