# Supplementary material for: Contribution of Extracranial Injuries to GOSE Scores after Traumatic Brain Injury TBI: A TRACK-Traumatic Brain Injury Study
Source: J Neurotrauma. 2025 Sep 12;42(17-18):1550–9. doi: 10.1089/neu.2024.0421 (PMC12677206; doi:10.1089/neu.2024.0421)
Supplement: Supplementary file 4 [file neu.2024.0421_supp_tables2.docx]

**eTable 2 - Mixed-Effects Linear Modelling of Difference in GOSE Scores.**

|  | **Primary** (Actual GOSE Diff.) | | **Sensitivity** (Percentile 0-100) | |
| --- | --- | --- | --- | --- |
| **Effect** | **B** (95% CI) | **p** | **B** (95% CI) | **p** |
| Intercept | 0.61  (0.56, 0.66) | ^<^.001 | 58.5  (57.2, 59.7) | ^<^.001 |
| Time (vs 2wk) | --- | ^<^.001 | --- | ^<^.001 |
| 3mo | -0.31  (-0.37, -0.24) | ^<^.001 | -6.8  (-8.3, -5.3) | ^<^.001 |
| 6mo | -0.43  (-0.50, -0.37) | ^<^.001 | -9.8  (-11.3, -8.3) | ^<^.001 |
| 12mo | -0.50  (-0.56, -0.44) | ^<^.001 | -11.7  (-13.2, -10.1) | ^<^.001 |
| Severity (vs Mild CT-) | --- | ^<^.001 | --- | ^<^.001 |
| Mild CT+ | -0.36  (-0.43, -0.28) | ^<^.001 | -8.2  (-10.0, -6.3) | ^<^.001 |
| Moderate/Severe | -0.49  (-0.58, -0.40) | ^<^.001 | -12.0  (-14.2, -9.9) | ^<^.001 |
| EC.Injury^1^ (vs not) | 0.82  (0.70, 0.94) | ^<^.001 | 15.6  (12.6, 18.5) | ^<^.001 |
| Time*Severity (vs 2wk Mild CT-) | --- | ^<^.001 | --- | ^<^.001 |
| 3mo Mild CT+ | 0.21  (0.11, 0.31) | ^<^.001 | 4.6  (2.2, 6.9) | ^<^.001 |
| 3mo Moderate/Severe | 0.23  (0.12, 0.34) | ^<^.001 | 5.0  (2.3, 7.6) | ^<^.001 |
| 6mo Mild CT+ | 0.31  (0.22, 0.41) | ^<^.001 | 6.6  (4.2, 8.9) | ^<^.001 |
| 6mo Moderate/Severe | 0.37  (0.26, 0.48) | ^<^.001 | 7.9  (5.3, 10.6) | ^<^.001 |
| 12mo Mild CT+ | 0.33  (0.24, 0.43) | ^<^.001 | 7.4  (5.0, 9.7) | ^<^.001 |
| 12mo Moderate/Severe | 0.42  (0.30, 0.53) | ^<^.001 | 9.9  (7.1, 12.6) | ^<^.001 |
| Time*EC.Injury (vs 2wk) | --- | ^<^.001 | --- | ^<^.001 |
| 3mo | -0.37  (-0.52, -0.22) | ^<^.001 | -4.2  (-7.8, -0.5) | .025 |
| 6mo | -0.69  (-0.84, -0.54) | ^<^.001 | -10.1  (-13.7, -6.5) | ^<^.001 |
| 12mo | -0.68  (-0.83, -0.53) | ^<^.001 | -10.8  (-14.5, -7.2) | ^<^.001 |
| Severity*EC.Injury (vs Mild CT-) | --- | .001 | --- | ^<^.001 |
| Mild CT+ | -0.12  (-0.32, 0.08) | .251 | 1.9  (-2.9, 6.7) | .441 |
| Moderate/Severe | -0.70  (-0.88, -0.53) | ^<^.001 | -14.1  (-18.3, -9.8) | ^<^.001 |
| Time*Severity*EC.Injury | --- | ^<^.001 | --- | ^<^.001 |
| Time Effect within Mild CT- no SECI^1^ | --- | ^<^.001 | --- | ^<^.001 |
| Time Effect within Mild CT+ no SECI^1^ | --- | ^<^.001 | --- | ^<^.001 |
| Time Effect within Mod/Sev no SECI^1^ | --- | .003 | --- | .007 |
| Time Effect within Mild CT- w/SECI^1^ | --- | ^<^.001 | --- | ^<^.001 |
| Time Effect within Mild CT+ w/SECI^1^ | --- | ^<^.001 | --- | ^<^.001 |
| Time Effect within Mod/Sev w/SECI^1^ | --- | .580 | --- | .390 |

^1^ Serious Extracranial Injury
