# Supplementary material for: Contribution of Extracranial Injuries to GOSE Scores after Traumatic Brain Injury TBI: A TRACK-Traumatic Brain Injury Study
Source: J Neurotrauma. 2025 Sep 12;42(17-18):1550–9. doi: 10.1089/neu.2024.0421 (PMC12677206; doi:10.1089/neu.2024.0421)
Supplement: Supplementary file 5 [file neu.2024.0421_supp_tables3.docx]

**eTable 3. Mean difference between GOSE-TBI and GOSE-All scores by TBI severity, extracranial injury seriousness and time based on mixed effects models^1^.**

|  | **No Serious Extracranial Injuries** | | | **With Serious Extracranial Injuries**^1^ | | |
| --- | --- | --- | --- | --- | --- | --- |
|  | **Mild CT-** | **Mild CT+** | **Mod/Sev** | **Mild CT-** | **Mild CT+** | **Mod/Sev** |
|  | GCS 13-15 | GCS 13-15 | GCS 3-12 | GCS 13-15 | GCS 13-15 | GCS 3-12 |
| 2 weeks | 0.61 | 0.26 | 0.12 | 1.43 | 0.96 | 0.24 |
| 3 months | 0.31 | 0.16 | 0.05 | 0.76 | 0.43 | 0.25 |
| 6 months | 0.18 | 0.13 | 0.06 | 0.30 | 0.27 | 0.23 |
| 12 months | 0.11 | 0.09 | 0.04 | 0.25 | 0.17 | 0.16 |

^1^Serious Extracranial Injury defined as Worst Extracranial AIS≥3
